# Supplementary material for: Data science competition for cross-site individual tree species identification from airborne remote sensing data
Source: PeerJ. 2023 Dec 21;11:e16578. doi: 10.7717/peerj.16578 (PMC10749090; doi:10.7717/peerj.16578)
Supplement: Supplemental Information 1 — Additional information about the Individual Tree Crown (ITC) and field data provided with the 2020 competition. [file peerj-11-16578-s001.docx]

**Appendix A**

This document provides additional information about the Individual Tree Crown (ITC) and field data provided with the 2020 competition.

# **NEON data products**

**Table A1. Data products from the National Ecological Observatory Network (NEON) used in the competition.**

| Data product | Description and links to NEON data product information | Spatial resolution | Data format provided to participants |
| --- | --- | --- | --- |
| High-resolution orthorectified camera imagery (RGB) | Raster data of the reflected energy from the surface as 3 bands representing the red, green, and blue portions of the spectrum. NEON data product ID: [DP1.30010.001](https://data.neonscience.org/data-product-view?dpCode=DP1.30010.001) | 10 cm^2^ | GeoTiff (.tiff) |
| Discrete return LiDAR point cloud (LAS) | Point cloud data of values in the X,Y, Z direction of the height of surface features and the ground. NEON data product ID: [DP1.30003.001](https://data.neonscience.org/data-product-view?dpCode=DP1.30003.001) | 6 points per m^2^ | LASer point cloud (.las) |
| LiDAR canopy height model (CHM) | Raster data containing the height of the top of the vegetation canopy. The CHM is a product of the LiDAR point cloud data. NEON data product ID: [DP3.30015.001](https://data.neonscience.org/data-product-view?dpCode=DP3.30015.001) | 1 m^2^ | GeoTiff (.tiff) |
| Mosaic  hyperspectral surface reflectance (HSI) | Raster data of reflected energy from the surface as 426 5-nm wide wavelength bands from 380-2510 nm. NEON data product ID: [DP3.30006.001](https://data.neonscience.org/data-products/DP3.30006.001) | 1 m^2^ | GeoTiff (.tiff) |
| Field measurements | Attributes of individuals measured in the field as part of the Woody Plant Vegetation Structure sampling protocol. NEON data product ID: [DP1.10098.001](https://data.neonscience.org/data-products/DP1.10098.001). | Individual tree | Comma separated value (.csv) |
| Individual tree crown (ITC) boundaries | 2-dimensional rectangular bounding boxes that geographically defines an ITC. Data are not standard NEON data products and were generated by the research group. | Individual tree | Esri Shapefile or .csv file with WKT format |

# **Location of Individual Tree Crown data**

#
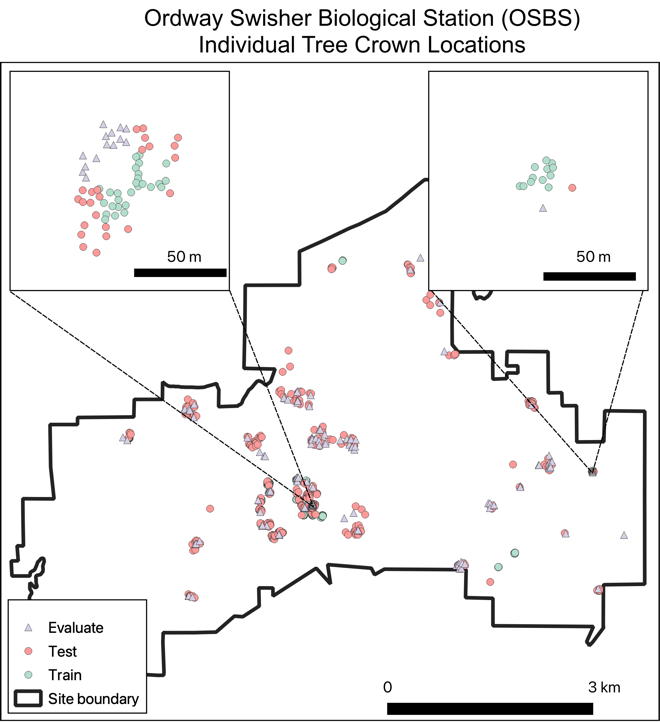

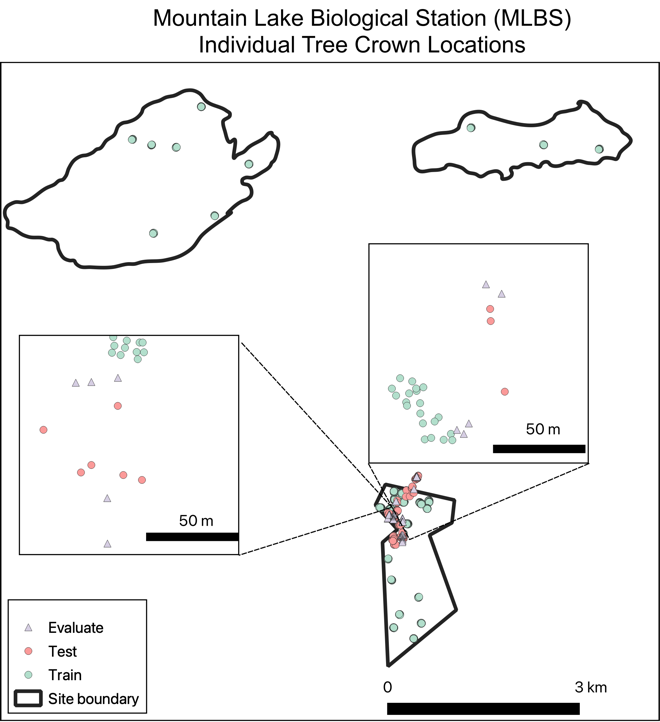


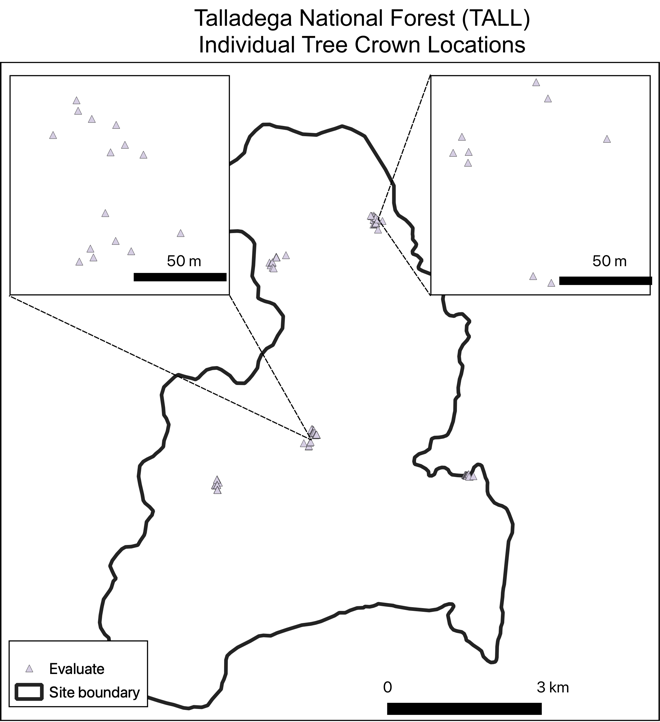


**Figure A1. Locations of Individual Tree Crowns (ITC) at each site**. ITCs are split at the NEON plot-level into test and train samples and provided to competition participants. Evaluation crowns were not provided to participants and occur outside the NEON plots. ITC are displayed here as points for visualization purposes but the data were provided as bounding boxes.

# **Individual Tree Crown (ITC) data**

**Field ITCs** were generated by members of the research team by visiting each NEON site and directly mapping ITCs in the remote sensing (RS) data while in the field (Table A2). RS data was loaded onto tablet computers that were equipped with GPS receivers. While in the field, researchers digitized crown boundaries based on the location, size, and shape of the crown seen in the field onto the RS data. Complete information for how the field ITC polygon data were generated are documented in Graves et al. 2018 (<https://peerj.com/preprints/27182/>).

Field ITCs were originally delineated as polygons to precisely match the irregular shape of each crown. For the competition, these polygons were converted to bounding boxes to capture the maximum boundary of the crowns in the North/South and East/West directions (Fig. A2). Field ITCs were collected in 2015 on remote sensing data from 2014, or collected in 2016 on 2015 data. The field ITCs were verified in the 2018 and 2019 imagery that are used in this competition. Any differences between the ITCs and the most recent imagery were corrected by shifting and or adjusting the dimensions of the ITC to match the position of the crown in the imagery. If differences could not be resolved, ITCs were removed from the dataset.

The field ITCs are considered the most accurate validation data and are used as the test data for the classification task. Species labels were generated by identification in the field or by collecting a voucher sample to be identified in an herbarium by a trained botanist. For individuals that could not be identified to species in the field or where a voucher could not be collected, the individual was identified to its genus category. A genus label was used for 43 individuals in the test data (7% of test data) and primarily for species of *Carya* and *Quercus.*

**Table A2**. Summary of the two types of ITC data used in the competition

| ITC type | field | image-only |
| --- | --- | --- |
| How they were generated | Manual delineation of crowns in the field directly on RS data. Species information from field or voucher identification. | Manual delineation of crowns using the full set of remote sensing data, but with no field visit. Species information from NEON vegetation structure data |
| How they are used in the competition | test data | train data |
| Association with NEON plots located within NEON sites | Are not located in NEON sampling plots. | Are located within NEON sampling plots and directly associated with NEON vegetation structure data. |


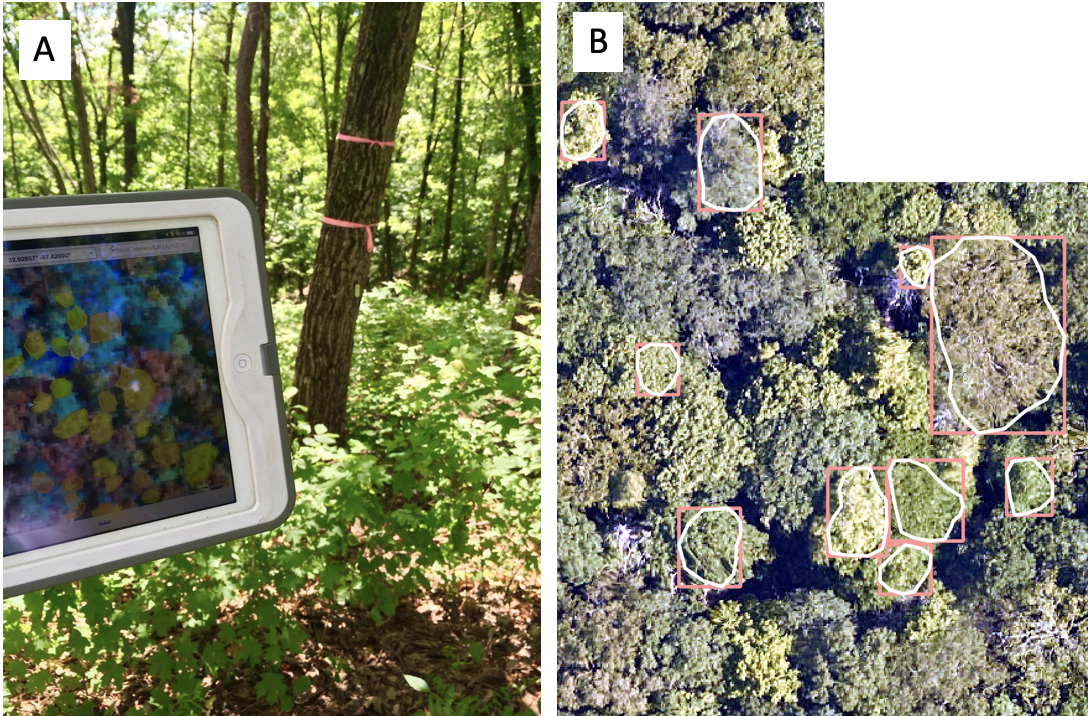


**Figure A2. Process and examples for generating field Individual Tree Crowns (ITCs)**. A) Tablet computer used for digitizing tree crowns in remote sensing data while in the field. B) Examples of ITCs on 2019 high-resolution RGB camera remote sensing data. Crown polygons (white) were mapped in the field and converted to bounding box extents (pink) to use as test data for the competition. Horizontal distance in this image spans 40 meters.

**Image-only ITCs** were generated by members of the research team by identifying tree crown boundaries using all available NEON RS data (Table A2). These ITCs were based on viewing lidar, RGB and hyperspectral images overlaid with point locations of stems and their attributes from NEON, and using expert knowledge to visually estimate the boundaries of individual tree crowns (Fig. A3). The field sites were not visited to generate image-only ITCs.

The image-only ITCs are considered by the research team to be the best available data that can be generated without observing individual trees in the field. Image-only ITCs are used to define the crown boundaries for the classification task. The species labels for image-only ITCs were generated by combining the ITC bounding box with NEON vegetation structure data (see next section). Using stem locations, crown sizes, and canopy position data from the NEON vegetation structure data, tree stems were matched to bounding boxes to provide species labels to each bounding box. Since all boxes needed to be assigned with an identifier, crowns not corresponding to any individual tree in the NEON vegetation structure were assigned with an arbitrary unique ID. This means that some ITCs do not have attribute data in the field data table.

Complete information for how the image-only ITCs were generated and assessment of the accuracy among expert digitizers is documented in Weinstein et al. (2020).


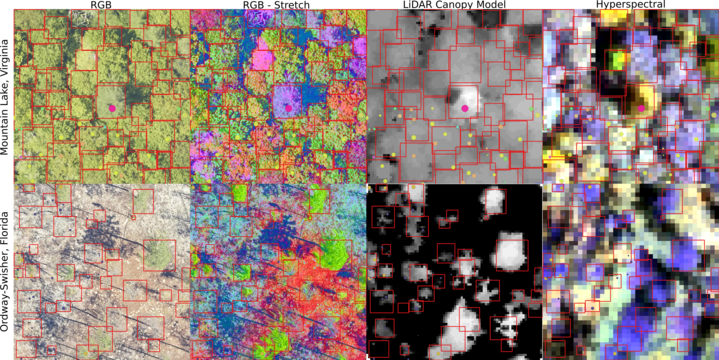


**Figure A3. Example of image-only ITC delineations (red boxes) on various remote sensing products for 2 NEON sites**. These remote sensing products were used to aid in the manual delineation of ITCs. Full details for how the image-only ITC delineations were created is in Weinstein et al. (2020)

# **NEON field data**

Field data are collected by the NEON Terrestrial Observation System (TOS) personnel. The data are collected in permanent plots that are repeatedly sampled. There are two types of plots associated with different field sampling objectives within NEON; the distributed plots that are spatially distributed throughout the NEON site and stratified by land cover type to capture the diversity of ecosystem types within each site; the tower plots, that are located within the airshed of the flux-tower. Data from both types of plots were used.

The field data used in this competition are from the Woody Plant Vegetation Structure sampling protocol (NEON 2020, protocol [DP1.10098.001](https://data.neonscience.org/data-products/DP1.10098.001)). The vegetation structure data was compiled using the NEONderive repository developed by members of the IDTReeS research team (<https://github.com/MarconiS/NEONderive>). The tabular data were provided as a comma separated value (csv) files that contained information on individual tree identifiers, location of trees relative to sampling locations (i.e. distance and azimuth from a central location), species and genus labels, and measures of relevant structural attributes (Table A3). The field attribute that was directly used in this competition is the taxonomic species information, described by the scientific name and the taxonomic identification code (Table A4).

**Table A3. Field data used in the data science evaluation.** The data provided were a subset of the full set of standard data collection by the NEON Terrestrial Observatory System. These attribute names and descriptions were also provided with the data.

| fieldName | description |
| --- | --- |
| indvdID | Domain-level unique identifier for an individual: NEON.MOD.D##.######. |
| siteID | NEON site code. Site codes used in this dataset are MLBS, OSBS, and TALL. |
| taxonID | Species code, based on one or more sources |
| scientificName | Scientific name, associated with the taxonID. This is the name of the lowest level taxonomic rank that can be determined |
| taxonRank | The lowest level taxonomic rank that can be determined for the individual or specimen |
| utmZone | UTM zone |
| nlcdClass | National Land Cover Database Vegetation Type Name |
| elevation | Elevation (in meters) above sea level |
| growthForm | The growth form classification: single-bole tree, multi-bole tree |
| plantStatus | Physical status of individual: live, dead, lost |
| stemDiameter | Cross-sectional stem diameter |
| height | Highest point of an individual or average height of a patch |
| maxCrownDiameter | Maximum crown diameter of the individual or patch |
| ninetyCrownDiameter | Crown diameter perpendicular to maxDiameter |
| canopyPosition | Vertical status of an individual relative to its neighbors |

**Table A4. Taxonomic species information for species classes used in the classification task and the sites at which they occurred.** The taxonomic species information is described by its scientific name, which includes a genus and species classification. To simplify the taxonomic species information, each scientific name was simplified to its unique taxonomic identification code. An “X” in the final column means the species was present in either the test or the train data at Ordway Swisher Biological Station (OSBS), Mountain Lake Biological Station (MLBS), or Talladega National Forest (TALL).

| taxonID | Scientific name | OSBS | MLBS | TALL |
| --- | --- | --- | --- | --- |
| ACER | *Acer sp.* |  | X |  |
| ACLE | *Acer leucoderme* |  |  | X |
| ACPE | *Acer pensylvanicum L.* |  | X |  |
| ACRU | *Acer rubrum L.* | X | X | X |
| ACSA3 | *Acer saccharum Marshall* |  | X |  |
| AMLA | *Amelanchier laevis Wiegand* |  | X |  |
| BETUL | *Betula sp.* |  | X |  |
| CACA | *Carpinus caroliniana* |  |  | X |
| CAGL8 | *Carya glabra (Mill.) Sweet* | X | X |  |
| CAOV | *Carya ovata* |  | X |  |
| CAPA | *Carya pallida* |  |  | X |
| CARYA | *Carya sp.* |  |  | X |
| CATO6 | *Carya tomentosa (Lam.) Nutt.* |  | X |  |
| DIVI | *Diospyros virginiana* | X |  | X |
| FAGR | *Fagus grandifolia Ehrh.* |  | X | X |
| FRPE | *Fraxinus pennsylvanica* |  |  | X |
| GOLA | *Gordonia lasianthus (L.) Ellis* | X |  |  |
| LIST | *Liquidambar styraciflua* | X |  | X |
| LITU | *Liriodendron tulipifera L.* |  | X | X |
| LYLU3 | *Lyonia lucida (Lam.) K. Koch* | X |  |  |
| MAAC | *Magnolia acuminata* |  | X |  |
| MAGNO | *Magnolia sp.* |  | X |  |
| MAGR | *Magnolia grandiflora* | X |  |  |
| MAMA | *Magnolia macrophylla* |  |  | X |
| MAVI | *Magnolia virginiana* | X |  | X |
| NYBI | *Nyssa biflora Walter* | X |  |  |
| NYSY | *Nyssa sylvatica Marshall* | X | X | X |
| OXYDE | *Oxydendrum sp.* |  | X |  |
| PEPA37 | *Persea palustris (Raf.) Sarg.* | X |  |  |
| PICL | *Pinus clausa* | X |  |  |
| PIEC | *Pinus echinata* |  |  | X |
| PIEL | *Pinus elliottii Engelm.* | X |  |  |
| PINUS | *Pinus sp.* | X | X |  |
| PIPA2 | *Pinus palustris Mill.* | X |  | X |
| PIST | *Pinus strobus* |  | X |  |
| PITA | *Pinus taeda L.* | X | X | X |
| PRSE2 | *Prunus serotina Ehrh.* |  | X |  |
| PRUNUS | *Prunus sp.* | X | X |  |
| QUAL | *Quercus alba L.* |  | X | X |
| QUCO2 | *Quercus coccinea* |  | X |  |
| QUERC | *Quercus sp.* |  | X | X |
| QUFA | *Quercus falcata* |  |  | X |
| QUGE2 | *Quercus geminata Small* | X |  |  |
| QUHE2 | *Quercus hemisphaerica W. Bartram ex Willd.* | X |  |  |
| QULA2 | *Quercus laevis Walter* | X |  |  |
| QULA3 | *Quercus laurifolia Michx.* | X |  |  |
| QUMO4 | *Quercus montana Willd.* |  | X | X |
| QUNI | *Quercus nigra L.* | X |  | X |
| QURU | *Quercus rubra L.* |  | X |  |
| QUVI | *Quercus virginiana* | X |  |  |
| ROPS | *Robinia pseudoacacia L.* |  | X |  |
| TSCA | *Tsuga canadensis (L.) Carriere* |  | X |  |

# **References**

Graves, S., Gearhart, J., Caughlin, T.T. and Bohlman, S., 2018. A digital mapping method for linking high-resolution remote sensing images to individual tree crowns. PeerJ Preprints, 6, p.e27182v1. <https://doi.org/10.7287/peerj.preprints.27182v1>

NEON (National Ecological Observatory Network). High-resolution orthorectified camera imagery (DP1.30010.001). https://data.neonscience.org (accessed March 4, 2021)

NEON (National Ecological Observatory Network). Discrete return LiDAR point cloud (DP1.30003.001). https://data.neonscience.org (accessed March 4, 2020)

NEON (National Ecological Observatory Network). Ecosystem structure (DP3.30015.001). https://data.neonscience.org (accessed March 4, 2020)

NEON (National Ecological Observatory Network). Woody plant vegetation structure (DP1.10098.001). https://data.neonscience.org (accessed March 4, 2020)

NEON (National Ecological Observatory Network). Spectrometer orthorectified surface directional reflectance - mosaic (DP3.30006.001). https://data.neonscience.org (accessed March 4, 2020)

Weinstein, B., Graves, S., Marconi, S., Singh, A., Zare, A., Stewart, D., Bohlman, S. and White, E.P., 2020. A benchmark dataset for individual tree crown delineation in co-registered airborne RGB, LiDAR and hyperspectral imagery from the National Ecological Observation Network. bioRxiv.
