# Supplementary material for: Data science competition for cross-site individual tree species identification from airborne remote sensing data
Source: PeerJ. 2023 Dec 21;11:e16578. doi: 10.7717/peerj.16578 (PMC10749090; doi:10.7717/peerj.16578)
Supplement: Supplemental Information 2 — The methods used by participating teams for the classification task. This also includes confusion matrices for the classification methods for each team. See the published articles for full details on the methods for some teams (Table B1). [file peerj-11-16578-s002.docx]

**Appendix B**

Team methods and results

The original intent of the competition was for individual teams to submit short methods and results papers describing the approaches and performance of their own methods. However, due to the COVID-19 pandemic this became untenable for most teams. This document describes the methods used by participating teams for the classification task. It also includes confusion matrices for the classification methods for each team. See the published papers for full details on the methods for some teams (Table B1).

**Table B1. List of teams that participated in the competition**. Teams for the baseline method was the winning team from the 2017 competition (Marconi et al. 2019). The baseline team did not participate in this competition and is not included in this appendix, but their methods were implemented on the data from this competition to compare their performance.

| Team name | Institution | Country | Paper reference |
| --- | --- | --- | --- |
| Fujitsu Satellite | Fujitsu Laboratories | Japan | - |
| Jeepers Treepers | University of Colorado Boulder | USA | Scholl et al. 2021 |
| Más JALApeñoS | Yale University | USA | - |
| Intellisence_CAU | China Agricultural University | China | - |
| Stanford-CCB (baseline) | Stanford University | USA | Anderson 2018 |

# **Fujitsu Satellite team**

Our method has two stages of classification: point-level (pixel-level) and crown-level (Figure B1). In the point-level classification, each hyperspectral point is regarded as a training sample with a label. After training the point-level classifier with the labeled data, the classifier was used to extract features for each hyperspectral point. The features were then used in the crown-level classifier. A new concept of “HSI texture” was used in our method to make the classification of tree species more robust. The purpose of crown-level classification is to mine the HSI texture data and follows an approach similar to BoW (bag of words) in NLP (Zhang, 2010).


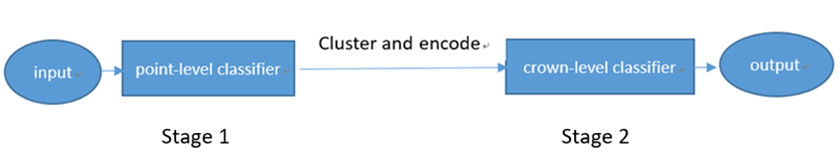


**Figure B1. Pipeline of the classification method.** The point-level classifier is trained and used as a feature extractor. All hyperspectral point features are clustered to get a codebook and each crown uses the codebook to generate a crown-level feature. Then a crown-level classifier is trained and used to generate the species class predictions.

We propose a point-level feature-based neural network to classify the tree species using hyperspectral data. The hyperspectral pixels are normalized by a min-max scaling method and fed into a pixel-level neural network to extract the pixel features. Min-max scaling uses a linear transformation to scale all hyperspectral bands to the same range (Fig. B2).


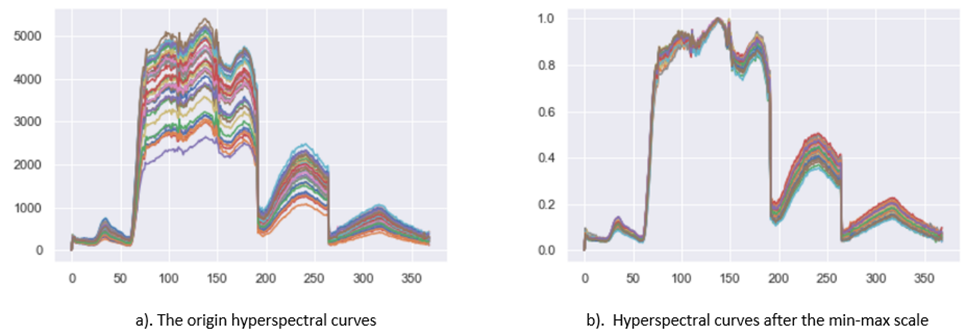


**Figure B2. An example of min-max scaling.** Left image shows the original hyperspectral curves with the X-axis the numeric band index and the Y-axis the reflectance value of each band (as percent reflectance scaled by 1000). Right image shows the hyperspectral curves after min-max scaling.

Figure B3 shows the structure of the pixel-level neural network classifier, which consists of four fully connected layers where N is the number of species. Rectified Linear Unit (ReLU) activation (Nair, 2010) is used after the fully connected layer except the last layer. The output dimensions of the fully connected layers are 2048, 4096, 2048, and N, respectively. Features from the third fully connected layer are extracted and used in the next stage.


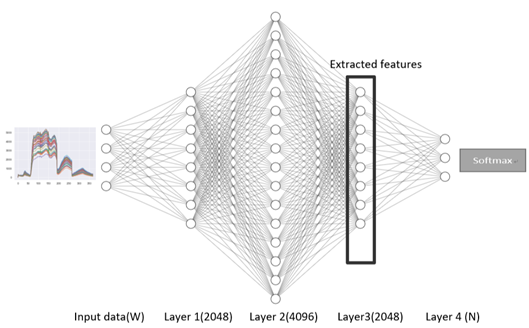


**Figure B3. Pixel-level neural network classifier.** The input data are the hyperspectral band values of each pixel in the crown. The network has three fully connected layers with node numbers 2048, 4096, and 2048 respectively. After the network is trained, the feature output of the 3^rd^ fully connected layer is taken as the input for the crown-level steps.

Mixup (Verma et al. 2019) and random spatial data augmentation are used for neural network training. Manifold mixup is used on features of randomly selected layers. Random spatial data augmentation is a new augmentation method proposed in our work. It is designed for using the spatial relationship of hyperspectral points. Weights are randomly assigned to the target point and its 8-neighbor points to generate a new point sample. This method takes 9 points as a cell (a center point and its eight neighborhoods, Fig. B4). New points are created by the nine points with randomly assigned weights (W1,W2,…W9) from a random gaussian distribution:

New point = (P1*W1 + P2*W2 + … , + P9*W9) / (W1 + W2 + … W9)


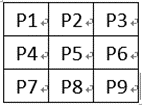


**Figure B4.** **A cell for random spatial data augmentation**.

In the training process, each point P has a possibility to be replaced. P is chosen as the center point in the cell and its 8 neighbors are used with random weights to calculate a new point.

For crown-level classification, we consider that in the image of a tree, not all hyperspectral points are absolutely similar. As a result of the scattering phenomenon, the distribution of point features in a crown is very important, which is why we define HSI texture for better robustness for the crown-level classification. For model training we extracted all pixel-level features and clustered the extracted features into L clusters. L should be several times larger than the number of species classes. We set L based on experience as 100 by the K-means method (Hartigan, 1979). The clusters were then encoded into indexes ranging from 0 to L-1. Each index is related to a clustered feature codebook. The clustered feature codebook is used to summarize all hyperspectral points into L classes. A tree species may include points from several classes composed under a certain ratio. That is what HSI texture means. With the L-dimension codebook, we can get L-dimension crown-level features for each crown following these steps:

1. Fetch the point-level feature (2048-dim) of the points in this crown.
2. For each point in this crown, find which group in our codebook is nearest to the feature and record the index.
3. For each point in this crown, the value of its index in the L-dim crown feature adds to 1.
4. A normalization is used. The L-dim feature is linearly scaled to make it sums to 1. Then we can get the crown-level features (L-dim).

The crown-level feature represents the geometry distribution of HSI texture for one individual tree, which is robust for the outliers. Finally, we trained a crown-level neural network to classify the individual tree crowns. The crown-level classifier was composed of two fully connected layers, the output number of which are 2048 and the number of tree species. ReLU activation was used after each fully connected layer followed by a softmax layer to calculate the probability of each sample.

**Fujitsu Satellite references**

Hartigan J A, Wong M A. Algorithm AS 136: A k-means clustering algorithm. Journal of the royal statistical society. series c (applied statistics), 1979, 28(1): 100-108.

Nair V, Hinton G E. Rectified linear units improve restricted boltzmann machines[C]//Icml. 2010.

Verma, V., Lamb, A., Beckham, C., Najafi, A., Mitliagkas, I., Lopez-Paz, D. and Bengio, Y., 2019, May. Manifold mixup: Better representations by interpolating hidden states. In International Conference on Machine Learning (pp. 6438-6447). PMLR.

Zhang Y, Jin R, Zhou Z H. Understanding bag-of-words model: a statistical framework. International Journal of Machine Learning and Cybernetics, 2010, 1(1): 43-52.


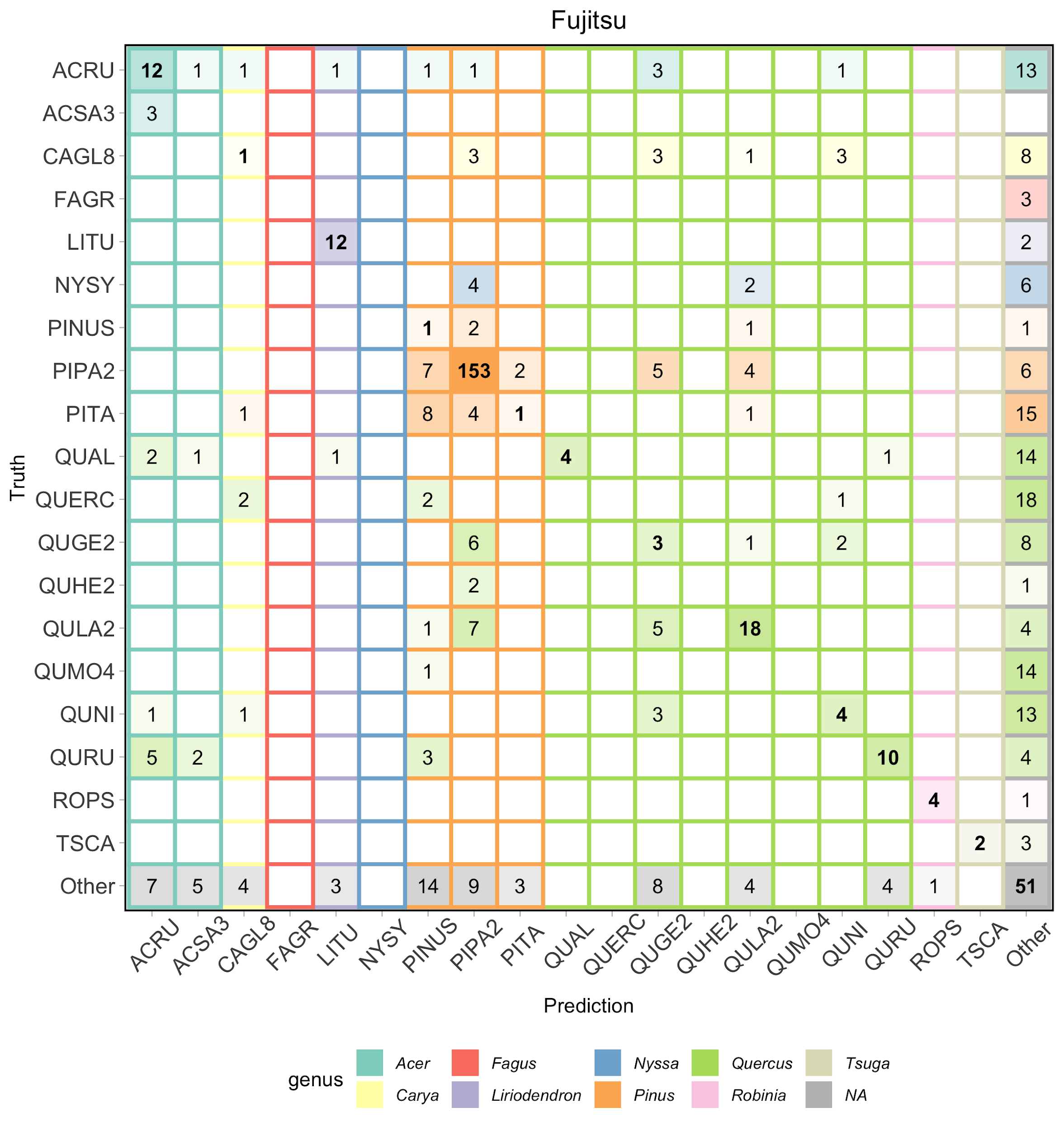


**Figure B5. Confusion matrix for the Fujitsu Satellite team.** Data from all three evaluation sites is included.

# **Jeepers Treepers team**

We used a two-stage neural network approach to integrate remote sensing data from all three sensors (RGB, hyperspectral, and lidar) and classify individual plant taxa (Scholl et al., 2021). First, we cropped a rectangular RGB image subset for each plant using the individual tree crown delineation polygons provided by the IDTReeS competition research group for the classification task. We then transformed the image subsets to have consistent dimensions of 224 x 224 pixels using a combination of cropping and resizing. Using these cropped RGB images, we trained a ResNet-34 convolutional neural network (He et al. 2016) pre-trained on the ImageNet dataset (Deng et al., 2009) to generate a taxon ID probability vector for each individual crown.

We concatenated this probability vector with the hyperspectral data extracted at crown centroids, along with pseudo-waveform data derived from the lidar point cloud. The pseudo-waveforms contain the density of discrete lidar returns within a series of height bins from a height of zero to the height above ground for any given tree, yielding a 1-dimensional profile representative of each tree’s vertical vegetation structure. This concatenated feature vector was passed to a second neural network that learned to fuse the RGB, hyperspectral, and lidar data together. We trained this multimodal neural network (Ngiam et al. 2011), also known as a fusion network (Goodfellow et al. 2016), minimizing a custom “soft F1” loss function to generate predictions that were robust to class imbalance in the training data. We then post-processed the predictions with a certainty less than 50% from the model by assigning 50% probability to an “other” category.


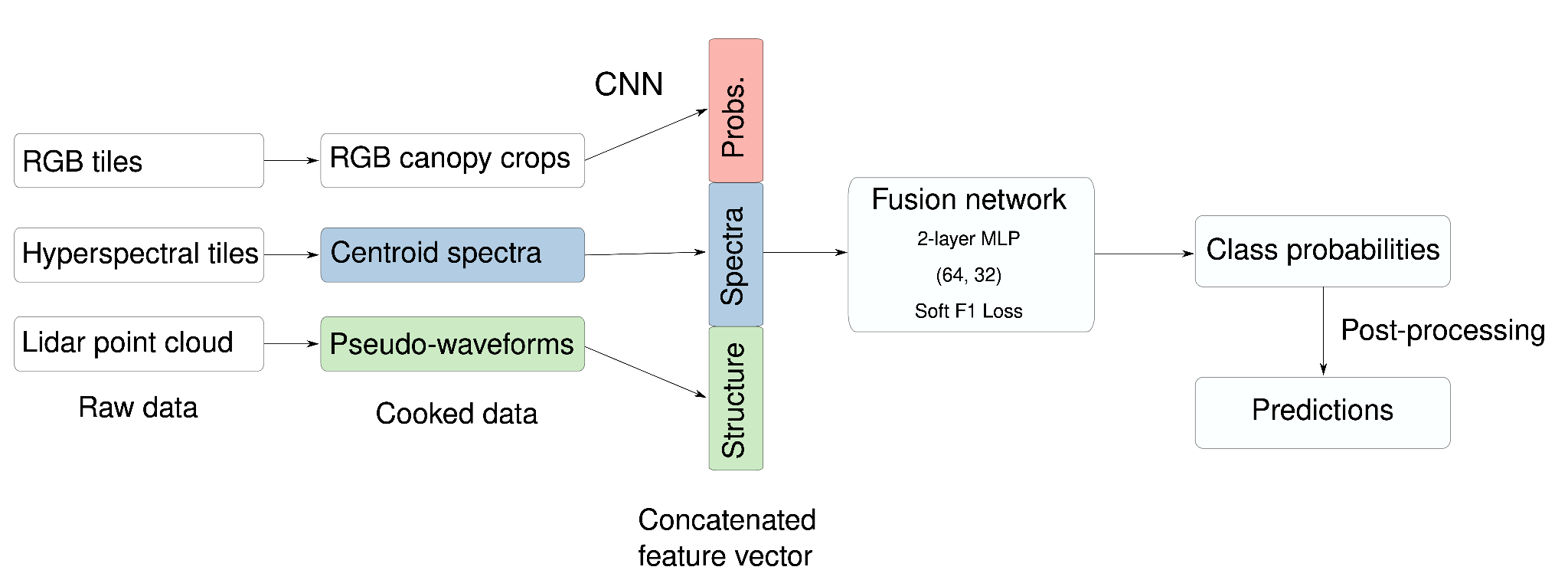


**Figure B6. Classification workflow used by the Jeepers Treepers team.** Raw remote sensing data products were process into formats that describe the spectral and structural characteristics for each individual plant canopy. A pre-trained Convolutional Neural Network (CNN) was used to estimate taxon probabilities using the RGB cropped canopy images, and combined these taxon probabilities with hyperspectral reflectance spectra and lidar-derived pseudo-waveforms into a concatenated feature vector. This feature vector was the input to the fusion network, a 2-layer multilayer perceptron (MLP) with two hidden layers (size 64 and 32) and trained using a custom “soft F1” loss function, to predict taxon class probabilities for each individual plant. We then post-processed the fusion network output to assign individuals to an “other” class when the classification confidence was less than 0.5. Finally, we produced predictions of taxon probabilities.


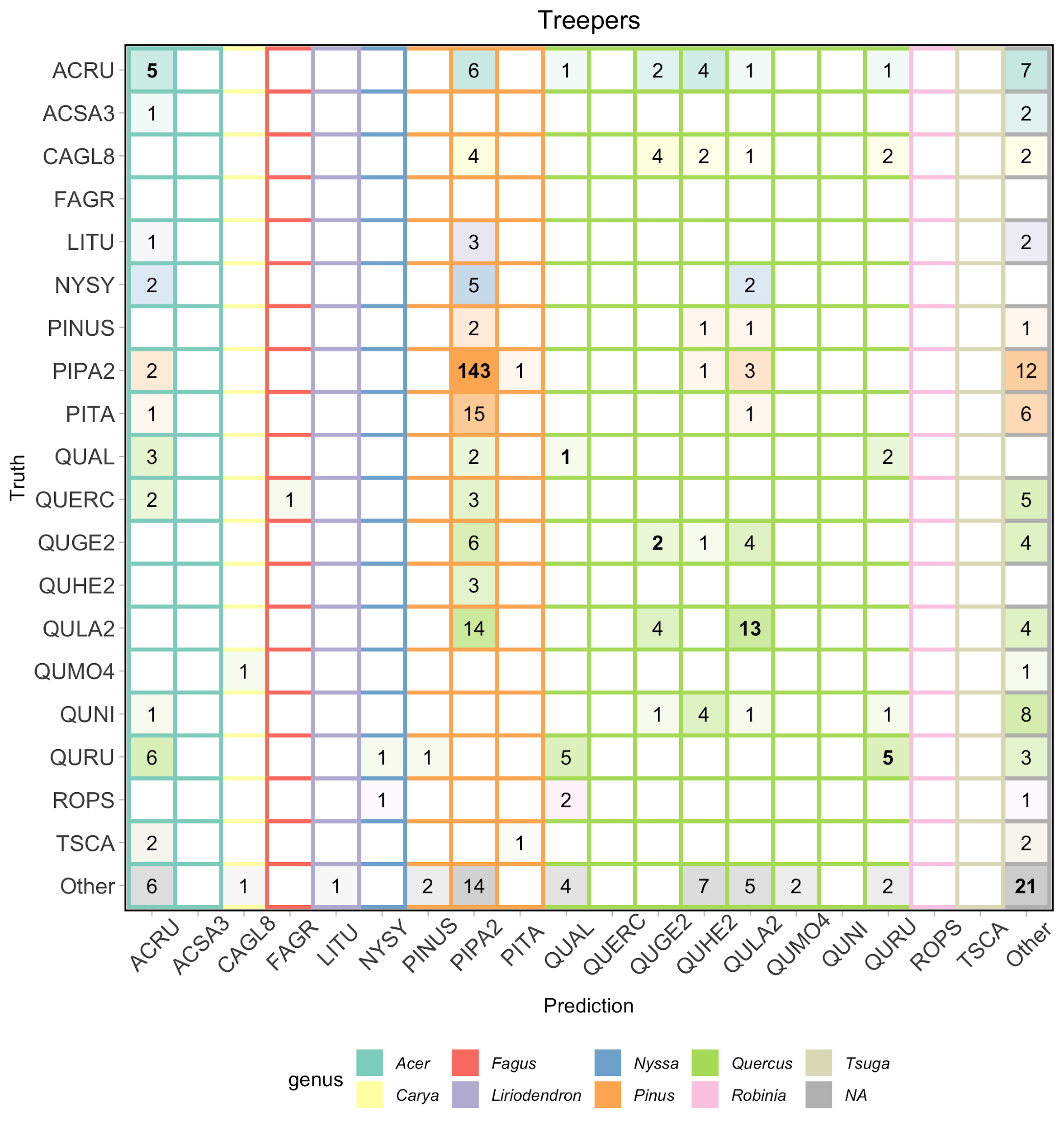


**Figure B7. Confusion matrix for the Jeepers Treepers team.** Data from all three evaluation sites is included.

**Jeepers Treepers references**

Deng J, Dong W, Socher R, Li L-J, Li K, Fei-Fei L. 2009. Imagenet: a large-scale hierarchical image database.

Goodfellow, I., Bengio, Y., Courville, A., and Bengio, Y. (2016). Deep learning, volume 1. MIT press Cambridge.

He, K., Zhang, X., Ren, S., and Sun, J. (2016). Deep residual learning for image recognition. In Proceedings of the IEEE conference on computer vision and pattern recognition, pages 770–778.

Ngiam, J., Khosla, A., Kim, M., Nam, J., Lee, H., and Ng, A. Y. (2011). Multimodal deep learning. In ICML.

Scholl VM, McGlinchy J, Price-Broncucia T, Balch JK, Joseph MB. 2021. Fusion neural networks for plant classification: learning to combine RGB, hyperspectral, and lidar data. PeerJ 9:e11790 <https://doi.org/10.7717/peerj.11790>

# **Más JALApeñoS team**

We used Extreme Gradient Boosting [(Chen & Guestrin, 2016)](https://paperpile.com/c/7oCoPX/TPPs) from the ‘xgboost’ R package for model fitting and prediction [(Chen et al., 2015)](https://paperpile.com/c/7oCoPX/tF0s). Our first data processing step was to crop the hyperspectral and canopy height rasters to the ITC bounding boxes of each individual tree in the training set. After removing instances where ITCs spanned 2 remote sensing images, we were left with 1,057 unique tree crowns (648 in MLBS and 409 in OSBS) in our training set. We applied an identical procedure to the testing set (71 ITCs in MLBS, 319 ITCs in OSBS, and 196 ITCs in TALL). Part of the classification challenge was to identify novel species the model was not trained on as ‘other’ species. To train the model to classify ‘other’ species, we labeled all individuals from species that had less than 3 individuals in the overall dataset as ‘other’ species, resulting in 12 individuals falling into this category. We determined the 3 individual threshold, as with many other parameters used in the modeling process, based on a partial grid search of parameter space (see below for details, Table B2).

Next, we removed all 1 m x 1 m pixels (n = 367 pixels) with negative values, which likely represent sensor errors. We then removed pixels (n = 2,197 pixels) with a canopy height ≤ 1 m, which may represent bare ground. Additionally, we removed individuals with < 5 pixels from the dataset so the model would not learn from individuals with small amounts of data.

To validate our model, we further split the training dataset into training and validation using a 70/30 split within each species. As such, approximately 70% of individuals within each species and their corresponding pixel-level data were randomly placed into the training set and the remaining 30% were used in the validation set. We conducted the training/validation split at the ITC level to avoid having one ITC being represented in both the training and validation set. In total, we had 23,155 pixels from 721 individuals in the training set and 9,919 pixels from 290 individuals in the validation set.

Following Anderson (2018), we filtered outlier pixels using a principal component analysis (PCA)-based method. First, we performed a PCA on the 369 hyperspectral bands on the pixels in the secondary training set (n = 23,155 pixels). We then removed a pixel if it was beyond a ± 5 standard deviation (SD) threshold in any of the first 50 principal components. We ran the same procedure on the validation pixels, except we used the PCA fit to the secondary training set rather than conducting a new PCA on the validation pixels. In total, this process filtered out 365 pixels from the secondary training set and 235 pixels from the validation set. We then PCA-transformed the hyperspectral imagery data in an attempt to isolate the potentially biologically-relevant components of the hyperspectral imagery that would describe the most variation in reflectance across different species in the dataset.

To determine the optimal parameter values for the hyperparameters in the Extreme Gradient Boosting model, as well as threshold values for data processing, we used a partial grid search approach. A grid search is the process of exploring potential parameter value combinations to determine the optimal parameter values. Because a full grid search that tested all 86,400 potential parameter combinations was computationally prohibitive, we used a partial grid search that explored a random subset of the potential range of parameter values (Table B2). The ranges of values chosen were arbitrary, but based on reasonable ranges from comparable models. In total, we fit 9,842 models in our partial grid search. For each randomly chosen parameter combination, we fit an Extreme Gradient Boosting model on the secondary training set (n = 23,155 pixels) and calculated the overall species classification accuracy of the fitted model on the validation set. Initial model runs were for 5,000 iterations, with the early stopping condition that the model fitting would stop if there was no improvement in model fit for 10 consecutive trees.

To calculate overall species classification accuracy, we used the fitted Gradient Boosting model to make predictions on each individual pixel in the validation and testing sets, resulting in a matrix containing the probability of each pixel belonging to each species in the training set. To create a species classification prediction at the ITC level, we averaged the species classification probability of each pixel within the ITC for an individual.

For species prediction on the testing set provided by the competition organizers, we followed data transformation procedures as described above, except that we did not filter out pixels based on the PCA (i.e., beyond a ± 5 standard deviation threshold in any of the first 50 principal components). We chose to not filter based on the PCA because filtering pixels based on the PCA in the training set may compromise the ability of the model to categorize species as ‘other’ for species that were not part of the training set.

To choose the final model, we limited our choice to the top 10 models in terms of highest prediction accuracy on the validation set in the partial grid search. The final model we chose to use on the testing set had the 5^th^ highest value of predictive accuracy on the validation set (0.662% vs. 0.668% for the highest value) and the lowest error in the training set (0.231% vs. 0.245% for next closest model) and 2^nd^ lowest error in the testing set (0.409% vs. 0.397% for lowest model). Altogether, the final model provided a balance between minimizing error and predictive accuracy. The parameter values for the final model are available in Table B2.

**Table B2. Parameter combinations used in partial grid search to determine optimal parameter values for species classification from hyperspectral data.** ‘Parameter’ is the description of the parameter being varied, ‘Xgboost parameter name’ is the parameter name used in the xgboost function, if applicable, ‘Range of values’ shows the range of parameter values explored in the partial grid search, and ‘Selected value’ shows the parameter value used in the final model.

| Parameter | Xgboost parameter name | Range of values | Selected value |
| --- | --- | --- | --- |
| Threshold for placing into ‘Other’ species | - | 3, 5 | 3 |
| Minimum canopy height | - | 1, 3 | 1 |
| Minimum pixel count | - | 0, 5 | 5 |
| SD threshold for filtering outlier pixels based on PCA | - | 3, 5 | 5 |
| Number of PCs to include in model | - | 25, 50, 100, 369 | 25 |
| Transform hyperspectral bands with PCA prior to model fitting? | - | True, False | TRUE |
| Learning rate | eta | 0.05, 0.1, 0.3, 0.5, 0.9 | 0.1 |
| Maximum tree depth | max_depth | 1, 4, 7, 10, 15 | 7 |
| Minimum child weight | min_child_weight | 1, 4, 7 | 7 |
| Subsample split of training data in xgboost model | subsample | 0.33, 0.66, 1.0 | 0.66 |
| Subsample split of prediction data in xgboost model | colsample_bytree | 0.2, 0.5, 1.0 | 0.5 |


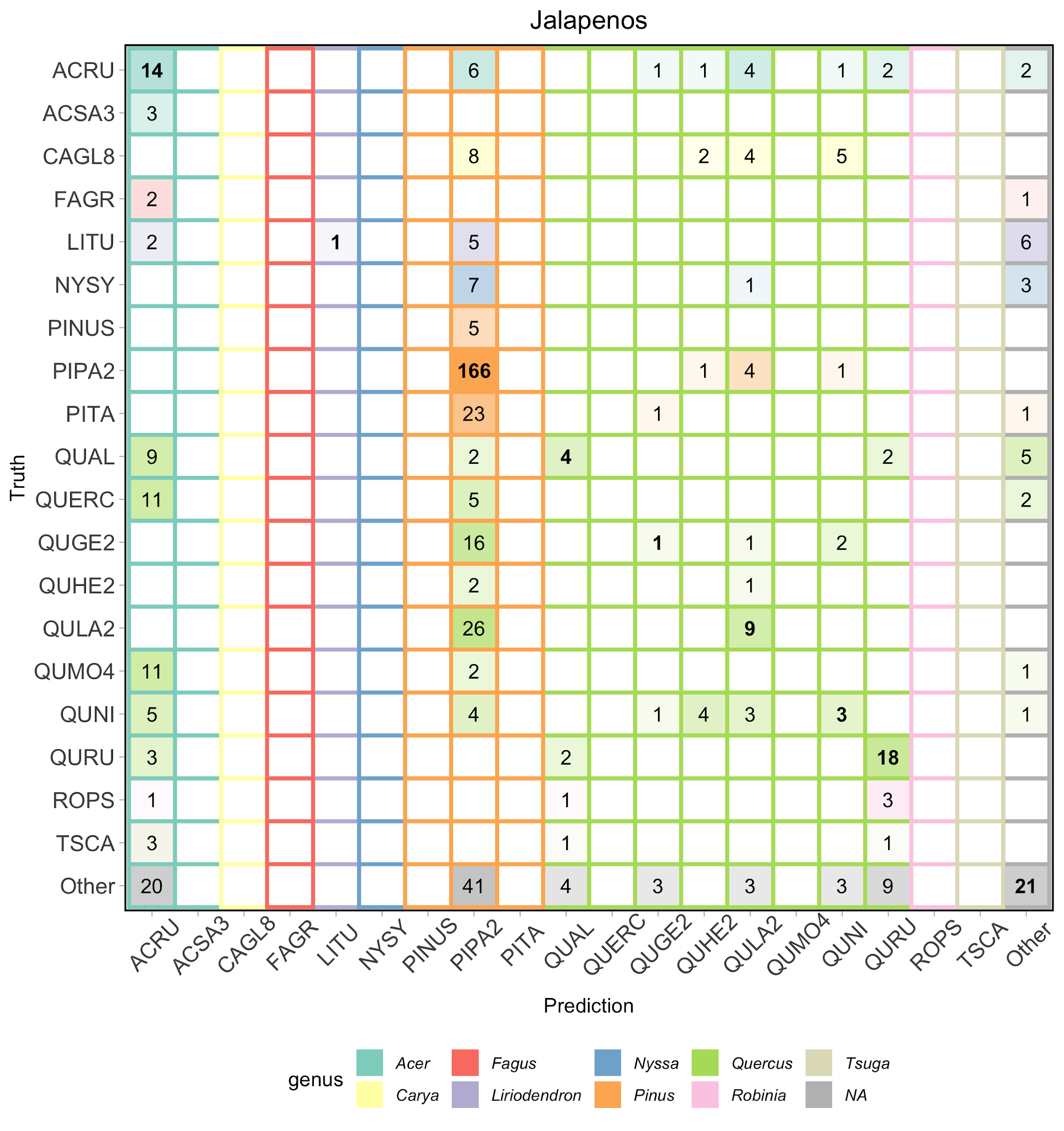


**Figure B8. Confusion matrix for the Más JALApeñoS team.** Data from all three evaluation sites is included.

**Más JALApeñoS References**

Anderson CB. 2018. The CCB-ID approach to tree species mapping with airborne imaging spectroscopy. PeerJ 6:e5666.

Chen T, Guestrin C. 2016. XGBoost: A Scalable Tree Boosting System. In: Proceedings of the 22nd ACM SIGKDD International Conference on Knowledge Discovery and Data Mining. KDD ’16. New York, NY, USA: Association for Computing Machinery, 785–794.

Chen T, He T, Benesty M, Khotilovich V, Tang Y. 2015. Xgboost: extreme gradient boosting. R package version 0. 4-2:1–4.

# **Intellisence CAU team**

The Intellisence CAU team was unable to provide additional details of their methods for the publication of these results. While this means that there is insufficient detail in the methods to replicate the analysis, we included their results for completeness.

We used a 1D-CNN to pixel values extracted from the hyperspectral data for species classification. The network architecture contains five layers with weights, including the input layer, one convolutional layer, one max pooling layer, the full connection layer, and the output layer. We initially followed two strategies to cope with the imbalanced data: (1) resampling the dataset, and (2) weighting the loss function. Based on the results from validation data, we chose the first strategy for making predictions on the held-out test dataset. To do so, we used a stratified random resampling strategy. For each species, we randomly selected between 400 and 600 individual trees, based on the original sample size of that species. Finally, we filtered outlier pixels using a canopy height model (CHM) threshold to separate pixels belonging to the crown from pixels belonging to the ground. This was particularly important for the OSBS site, where open crowns showed multiple ground pixels within the bounding boxes.


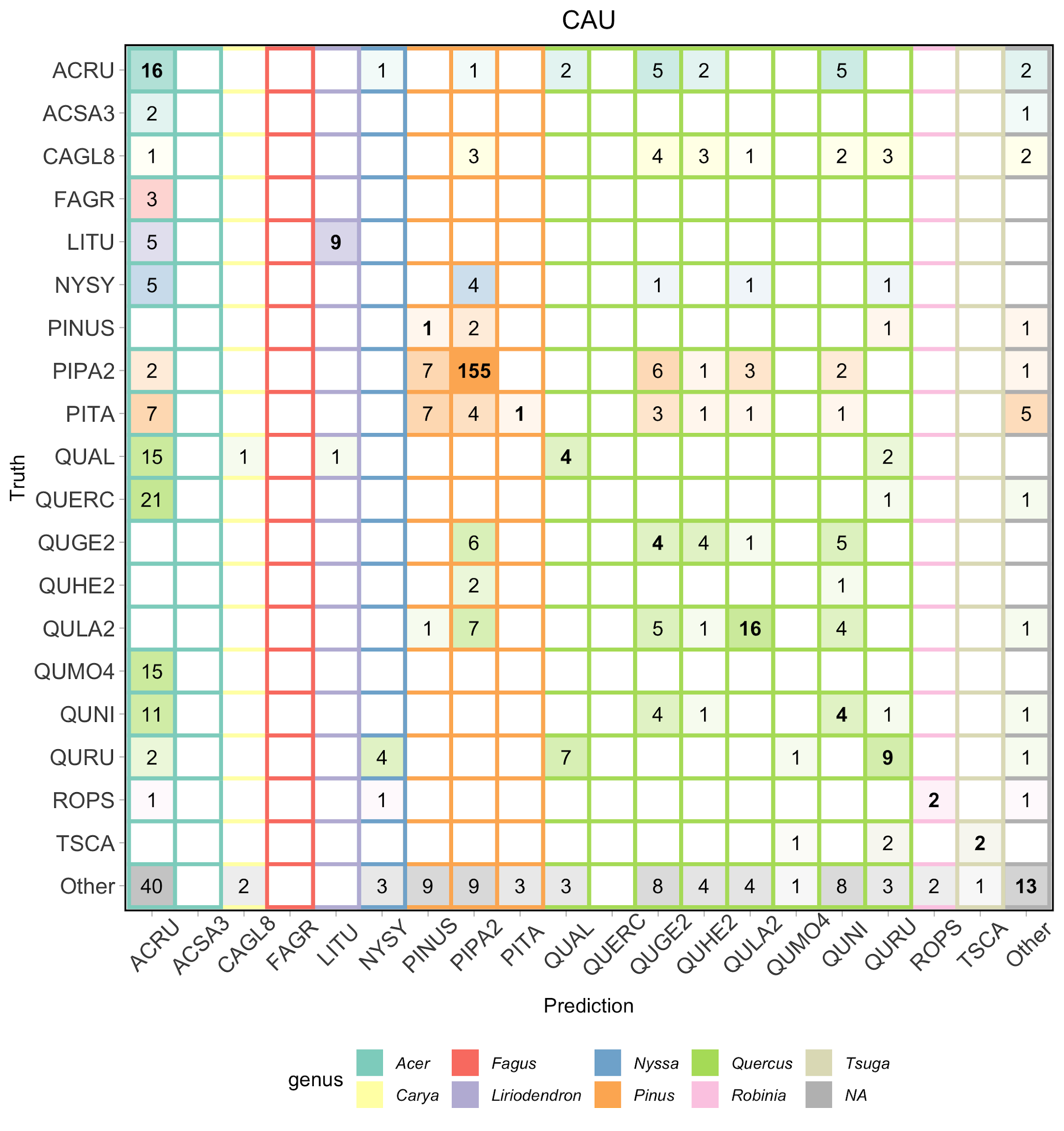


**Figure B9. Confusion matrix for the CAU team.** Data from all three evaluation sites is included.

**CAU references**

Shanmugam, D., Blalock, D., Balakrishnan, G., & Guttag, J. (2020). When and Why Test-Time Augmentation Works. arXiv preprint arXiv:2011.11156.
